# Correlations of disease severity and age with hematology parameter variations in patients with COVID‐19 pre‐ and post‐treatment

**DOI:** 10.1002/jcla.23609

**Published:** 2020-11-21

**Authors:** Juanying Liang, Shaoyun Nong, Liejun Jiang, Xiaowei Chi, Dewu Bi, Jun Cao, Lida Mo, Xiaolu Luo, Huayi Huang

**Affiliations:** ^1^ Department of Laboratory Medicine The Forth People's Hospital of Nanning City Nanning China; ^2^ Department of Laboratory Medicine Guangxi Nationalities Hospital Nanning China; ^3^ Department of Laboratory Medicine The People's Hospital of Guangxi Zhuang Autonomous Region Nanning China; ^4^ Department of Laboratory Medicine The Affiliated Hospital of Shandong University of Traditional Chinese Medicine Jinan China; ^5^ School of Medical Laboratory Youjiang Medical University for Nationalities Baise China; ^6^ Mindray North America Mahwah NJ USA; ^7^ Department of Surgical Oncology Roswell Park Comprehensive Cancer Center Buffalo NY USA

**Keywords:** Coronavirus Disease 2019, C‐reactive protein, neutrophil/lymphocyte ratio, red blood cell distribution width, T‐cell subsets

## Abstract

**Background:**

For better understanding of the pathological changes of COVID‐19, benefiting clinical management of the disease and the preparation for future waves of similar pandemics.

**Methods:**

Hematology parameters from a total of 52 cases of COVID‐19 admitted for treatment in a designated hospital were retrospectively analyzed. Data were analyzed by SPSS statistical software.

**Results:**

Pre‐treatment T‐cell subsets, total lymphocytes, red blood cell distribution width (RDW), eosinophils, and basophils were significantly lower than that of post‐treatment, while the inflammatory indexes neutrophils, neutrophil to lymphocyte ratio (NLR), and C‐reactive protein (CRP) levels, as well as red blood cell (RBC) and hemoglobin, were significantly reduced after treatment. The T‐cell subsets, total lymphocytes, and basophils in severely and critically ill patients were significantly lower than those in moderately ill patients. Neutrophils, NLR, eosinophils, procalcitonin (PCT), and CRP was significantly higher in severely and critically ill patients than in moderately ill patients. CD3+, CD8+, total lymphocytes, platelets, and basophils in patients older than 50 were lower than that of those younger than 50, while neutrophils, NLR, CRP, and RDW in patients older than 50 were higher than that of younger than 50. There was a positive correlation among prothrombin time (PT), alanine aminotransferase (ALT), and aspartate aminotransferase (AST) in severely and critically ill patients.

**Conclusions:**

T‐cell subsets, lymphocyte count, RDW, neutrophils, eosinophils, NLR, CRP, PT, ALT, and AST are important indicators in the management especially for severely and critically ill patients with COVID‐19.

## Graphical abstract

This work was to observe the correlation of the inflammatory indexes in the blood of COVID‐19 patients pre‐ and post‐ treatment for better understanding of the pathological changes of COVID‐19 benefiting clinical management of the disease and the preparation for future waves of similar pandemics.

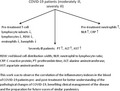

## 1 INTRODUCTION

The coronavirus disease 2019 (COVID‐19) pandemic caused by the novel coronavirus broke out in December of 2019 and has spread rapidly worldwide.^1^, ^2^, ^3^ At the beginning of the outbreak, clinical focus was emphasized on the manifestations and epidemiology together with computed tomography imaging of the patients,^4^, ^5^ and the diagnosis would then made by a positive nucleotides amplification result. However, various pathological damages have been found in different organs later on.^6^, ^7^, ^8^, ^9^ Accumulating evidence indicates that COVID‐19 is rather complex in pathophysiological changes with multi‐organ damage from the virus attack and the hyper‐reaction of the immune system. Elevation of cytokines and inflammatory reactive proteins in serum and in the alveolar of the lungs has been observed,^7^, ^10^, ^11^, ^12^ while lymphocytopenia and abnormality of T‐cell subsets were found in severely ill patients.^13^, ^14^ Neutrophil to lymphocyte ratio was reported to be a useful indicator in differentiating malign and benign thyroid nodules in clinical practice.^15^ The NLR was also useful in helping differentiate patients with ulcerative colitis from healthy controls.^16^ It also plays a role in thyroiditis and is associated with type 2 diabetes.^17^, ^18^ RDW is a marker of anisocytosis of the erythrocytes. Study found that it was helpful in the differentiation of thyroid nodules, in diagnosis of rheumatoid arthritis, lumbar disk pathologies, and in thyroiditis,^19^, ^20^, ^21^ while CRP is a universal inflammatory predictor which has been studied in many conditions.^22^ Recently found that NLR, RDW, and CRP were also involved in COVID‐19 and play an important role in the diagnosis and prognosis of the disease.^11^, ^14^, ^23^, ^24^, ^25^ Thus, laboratory findings are important in evaluating patient's condition and making treatment decision. We retrospectively analyzed the laboratory parameters categorized by pre‐ and post‐treatment, severity, and age of 52 cases of COVID‐19 whom were hospitalized in a designated hospital in Southern China for further understanding of the pathological changes of the disease and for aiding the clinical management of COVID‐19 in the future.

## 2 MATERIALS AND METHODS

### 2.1 Patients

A total of 52 COVID‐19 patients who were admitted for treatment in a designated hospital, the Forth Hospital of Nanning City, were analyzed retrospectively in this study from January 24, 2020, to March 2, 2020. Among them, 45 cases were moderately ill, 5 cases were severely ill, and 2 cases were critically ill, with age ranging from 3 months to 85 years old. For gender, 27 cases were male and 25 cases were female. The patients had manifestations such as fever, dry cough, feebleness, headache, shortness of breath, nasal congestion, runny nose, sore throat, muscle pain, diarrhea, myalgia,. Computed tomography scans showed patchy or ground‐glass like imaging of the lungs, indicating pneumonia. Diagnosis was made following the China Guidelines on the Diagnosis and Treatment of COVID‐19, Version 7. A diagnosis was confirmed by real‐time qPCR detection of virus nucleotides. Patients were categorized into moderately, severely, and critically ill groups based on the diagnostic criteria. In moderate cases, patients presented fever and respiratory syndromes, and imaging results displayed pneumonia patterns. Severely ill diagnosis was made if patients met any of the following criteria: (a) respiratory distress (respiratory rate ≥30/min); (b) resting finger oxygen saturation ≤93%; (c) arterial oxygen pressure (PO2)/ fraction of inspiration O2 (Fi O_2_) ≤300 mm Hg (1 mm Hg = 0.133 kPa). Critically ill diagnosis was made if patients met any of the following criteria: (a) respiratory failure requiring use of mechanical ventilation; (b) shock; (c) other organ failure which requires treatment in the intensive care unit (ICU). Following the above criteria, the 52 patients were diagnosed as 2 critically ill, 5 severely ill, and 45 moderately ill cases.

### 2.2 Basic treatment procedures

All patients including moderate, severely and critically ill patients were treated with the following basic procedures: (a) general auxiliary treatment; (b) anti‐virus treatment: lopinavir/ ritonavir and α‐interferon; (c) a formula of Traditional Chinese Medicine with adjustable doses based on the patients' conditions.

This study was approved by the Institute Review Board of the Forth Hospital of Nanning City for the collection of patient information.

### 2.3 Laboratory parameters

Hematology analysis for peripheral blood: routine hematology analysis for peripheral blood was performed on a Mindray BC‐6900 hematology analyzer (Mindray) and a Sysmex XN 9000 hematology analyzer (Sysmex). Fasting ethylenediaminetetraacetic acid (EDTA) anticoagulated blood sample was collected the morning following the patient's admission. The consistency evaluation between the mentioned two hematology analyzers was verified following the laboratory quality control procedures. White blood cell (WBC) count and differentiation, red blood cell (RBC), and indices were obtained alongside a scattergram and histogram in the hematology analysis.

Flow cytometry for T lymphocyte subset: Flow cytometry analysis was performed to analyze the T‐cell subsets using a BD (Becton, Dickinson and Company) FACSCalibur flow cytometer. Data were analyzed by MultiSET software. The assay was performed following standard operating procedures and manufacturer's instructions. Two milliliters of venous blood were collected using the EDTA anticoagulant blood collecting tube. The sample was mixed gently by overturning the sample tube several times to prevent clotting. The samples were delivered to the laboratory after collection and were analyzed within 6 hours at room temperature.

Immunofluorescent assay: C‐reactive protein (CRP) and procalcitonin (PCT) were analyzed using the blood sample from the hematology analysis immediately after finishing said analysis, and assays were performed on an FS‐112 immunofluorescent analyzer (Wondfo Biotech Co., LTD.) following the manufacturer's instructions and laboratory standards of procedure.

Serum alanine aminotransferase (ALT) and aspartate aminotransferase (AST) were analyzed on a HITACHI LABOSPECT008AS chemistry analyzer (HITACHI). Prothrombin time (PT) was analyzed on a STAGO STA‐R Evolution analyzer (Diagnostica Stago).

Reverse transcription quantitative polymerase chain reaction (RT‐qPCR): RT‐qPCR to detect SARS‐CoV‐2 was performed using the RNA template isolated from either the nasopharyngeal swab or secretions of the lower respiratory track. The nucleic acids were isolated on a SSNP‐2000A nucleic acid automatic isolation platform (Bioperfectus Technologies). The assay kit was provided by Sun Yat‐sen University DAAN Gene Co., Ltd. and Shanghai BioGerm Medical Biotechnology Co., Ltd. Thermo cycling was performed on an ABI 7500 thermo cycler (Applied Biosystems). Virus nucleosides detection results were defined as either positive or negative.

### 2.4 Statistical analysis

Data were analyzed using the SPSS version 18.0 software; paired samples *t* test, independent samples *t* test, or Mann‐Whitney *U* test were applied, and a *P* value <.05 was considered significant.

## 3 RESULTS

### 3.1 Hematological parameters of severely and critically ill patients

The 5 severely and 2 critically ill patients were older than patients in the moderately ill group (69.3 vs 40.4). The detailed information of the 5 severely and 2 critically ill patients was shown in Table 1A and B. In severely and critically ill patient group, the T‐cell subsets, and total lymphocytes were usually low, but WBC counts were roughly normal except for one patient who showed elevated WBC (11.5 × 10^9^/L). Neutrophils and monocytes were usually high as well. Serum PCT, ALT, AST, and PT values were high in 2 critically ill patients and 1 severely ill patient, and there was a positive correlation among PT, ALT, and AST in 1 severely and 2 critically ill patients. Nearly all 7 patients had high CRP levels. Eosinophils (EOS) and basophils (BASO) tended to be low in critically and severely ill patients (Table 1A and B). A description of the normal ranges of hematological parameters in the adult Chinese population is listed under Table 1.

**Table 1:** Hematology parameters of 7 severely and critically ill patients (pre‐treatment)

A													
Patient ID	Sex	Age	CD3+	CD4+	CD8+	CD4+/CD8+	WBC	Neu%	Lym%	Mon%	PCT	CRP	Condition
1	M	85	108	56	53	1.06	4.0	91.6	2.7	5.7	0.24	9.9	Critical
2	M	70	738	373	344	1.08	6.8	79.3	1.6	4.2	0.63	9.9	Critical
3	M	73	278	175	95	1.84	5.0	85.2	8.2	6.1	0.15	8.9	Severe
4	F	69	154	84	58	1.45	3.7	88.6	6.5	4.3	<0.1	<5	Severe
5	M	53	388	302	75	4.03	11.5	83.2	12.0	4.0	<0.1	58.3	Severe
6	F	66	1596	1002	570	1.76	3.8	70.2	23.7	4.8	<0.1	44.8	Severe
7	F	69	165	79	77	1.03	7.7	63.7	19.9	14.3	0.10	44.9	Severe

B												
Patient ID	Sex	Age	Hb	RBC	RBC RDW S	PLT	PT	EOS%	BASO%	ALT	AST	Condition
1	M	85	155	5.41	47.8	74.0	200.0	0.0	0.0	63.0	57.0	Critical
2	M	70	126	4.25	38.5	259.0	129.0	0.0	0.1	123.0	99.0	Critical
3	M	73	206	6.68	45.1	262.0	14.0	0.0	0.2	18.0	29.0	Severe
4	F	69	191	6.34	39.6	99.0	12.5	0.3	0.3	28.0	19.0	Severe
5	M	53	109	3.65	42.7	310.0	13.0	0.5	0.3	42.0	25.0	Severe
6	F	66	96	3.84	41.0	143.0	15.4	1.3	0.0	57.0	50.0	Severe
7	F	69	113	3.87	38.1	294.0	15.0	0.2	0.3	18.0	16.0	Severe

Note

Reference ranges of some hematologic parameters used in this study in adult Chinese population: WBC 4.0‐10.0 × 10^9^/L; neutrophils: 50%‐70% of WBC; lymphocytes: 20%‐40% of WBC; monocytes: 3%‐8% of WBC; eosinophils: 0.4%‐8%; basophils: 0%‐1%; hemoglobin: 120‐160 g/L (male), 110‐150 g/L (female); RBC: 4.0‐5.5 × 10^12^/L (male), 3.5‐5.0 × 10^12^/L (female); RBC RDW S: 35.0‐56.0; PLT: 100‐300 × 10^9^/L; PT: 11‐13 s; PCT: <0.1 µg/L; CRP: <5 mg/L; ALT: 5‐40 U/L; AST: 8‐40 U/L.

Abbreviations: CRP, C‐reactive protein; NLR, neutrophil to lymphocyte ratio; PCT, procalcitonin; RBC, red blood cell; WBC, White blood cell.

### 3.2 Comparison of pre‐ and post‐treatment hematological parameters in COVID‐19 patients

Statistical analysis results showed that pre‐treatment CD3+, CD4+, CD8+ T cells, total lymphocytes, RBC distribution width (RDW), eosinophils, and basophils were significantly lower than that of post‐treatment (*P* = .000, .000, .000, .012, .04, .000, and .001, respectively). While the inflammatory indexes neutrophils, neutrophil/lymphocyte ratio (NLR), and CRP were significantly higher in pre‐treatment than in post‐treatment (*P* = .004, .011, and .017, respectively). Hb and RBC were significantly reduced post‐treatment (*P* = .032, .026). The PLT increased, albeit not significantly, after treatment (*P* = .183) (Table 2).

**Table 2:** Comparison of hematology parameters pre‐ and post‐treatment in COVID‐19 patients (paired samples *t* test)

Parameter	Pre‐treatment	Post‐treatment	*P* value	Significance
CD3+	1006	1184	.000	Yes
CD4+	597.7	696.8	.000	Yes
CD8+	364.6	457.3	.000	Yes
CD4+/CD8+	1.789	1.589	.131	No
WBC	6.48	6.60	.701	No
Neutrophil%	67.03	62.20	.004	Yes
Lymphocyte%	24.03	27.57	.012	Yes
NLR	4.26	2.74	.011	Yes
Monocyte%	7.737	7.488	.623	No
Hb	136	128	.032	Yes
RBC	4.74	4.46	.026	Yes
RBC RDW‐S	39.6	41.8	.04	Yes
PLT	263	280	.183	No
Eosinophil%	1.3	3.1	.000	Yes
Basophil%	0.3	0.5	.001	Yes
PCT	0.1288	0.1181	.354	No
CRP	14.51	8.192	.017	Yes

Abbreviations: CRP, C‐reactive protein; NLR, neutrophil to lymphocyte ratio; PCT, procalcitonin; RBC, red blood cell; WBC, White blood cell.

### 3.3 Comparison of hematological parameters between moderately and severely and critically ill patients

The T‐cell subsets (CD3+, CD4+, CD8+), total lymphocytes, and basophils in severely and critically ill patients were significantly lower than those in moderately ill patients (*P* = .025, .048, .027, .006, and .046, respectively). Neutrophils, NLR, PCT, and CRP levels were significantly higher in severely and critically ill patients than in moderately ill patients (*P* = .005, .002, .049, and .002, respectively). The PLT in severely and critically ill patients was lower than in moderately ill patients; however, the differences were not statistically significant (Table 3).

**Table 3:** Comparison of pre‐treatment hematology parameters between moderate and severely and critically ill patients (independent samples *t* test or Mann‐Whitney *U* test)

Parameter	Moderate	Critical and severe	*P* value	Significance
CD3+	1085.76	489.57	.025	Yes
CD4+	644.69	295.86	.048	Yes
CD8+	393.09	181.71	.027	Yes
CD4+/CD8+	1.79	1.75	.901	No
WBC	6.54	6.07	.658	No
Neutrophil%	64.97	80.26	.005	Yes
Lymphocyte%	25.79	12.74	.006	Yes
NLR	3.23	10.85	.002	Yes
Monocyte%	7.98	6.20	.196	No
Hb	134.8	142.3	.896	No
RBC	4.7	4.9	.694	No
RBC RDW‐S	39.2	41.8	.087	No
PLT	271.6	205.9	.081	No
Eosinophil%	2.7	5.4	.006	Yes
Basophil%	0.3	0.2	.046	Yes
PCT	0.12	0.20	.049	Yes
CRP	8.89	34.37	.002	Yes

Abbreviations: CRP, C‐reactive protein; NLR, neutrophil to lymphocyte ratio; PCT, procalcitonin; RBC, red blood cell; WBC, White blood cell.

### 3.4 Comparison of hematological parameters between age under 50 years and age above 50 years old patients

CD3+, CD8+, total lymphocytes, platelets, and basophils in patients older than 50 years were significantly lower than that of younger than 50 years (*P* = .049, .018, .019, .010, and .039, respectively), while neutrophils, NLR ratio, CRP level, and RDW in patients older than 50 years were significantly higher than that of younger than 50 years (*P* = .0191, .015, .009, and .010, respectively) (Table 4).

**Table 4:** Comparison of pre‐treatment hematology parameters between ages under 50 and above 50 patient groups (independent samples *t* test or Mann‐Whitney *U* test)

Parameter	<50 y old	>50 y old	*P* value	Significance
CD3+	1120.38	722.13	.049	Yes
CD4+	654.84	456.87	.140	No
CD8+	413.70	243.60	.018	Yes
CD4+/CD8+	1.66	2.12	.086	No
WBC	6.68	5.98	.380	No
Neutrophil%	64.22	73.93	.019	Yes
Lymphocyte%	26.49	17.97	.019	Yes
NLR	3.15	6.98	.015	Yes
Monocyte%	8.05	6.97	.297	No
Hb	136	135	.864	No
RBC	4.8	4.5	.136	No
RBC RDW‐S	38.8	41.6	.010	Yes
PLT	284	211	.010	Yes
Eosinophil%	2.8	3.8	.173	No
Basophil%	0.3	0.2	.039	Yes
PCT	0.11	0.17	.204	No
CRP	6.28	27.47	.009	Yes

Abbreviations: CRP, C‐reactive protein; NLR, neutrophil to lymphocyte ratio; PCT, procalcitonin; RBC, red blood cell; WBC, White blood cell.

## 4 DISCUSSION

COVID‐19 is caused by infection of a coronavirus SARS‐CoV‐2 which was first characterized in December 2019 in Wuhan, China. The outbreak of SARS‐CoV‐2 has since spread rapidly and led to a worldwide pandemic.^1^, ^2^, ^3^ Due to limited knowledge of the virus's epidemiology and pathology, the mortality rate was high at the beginning of the outbreak. Subsequent management and treatment of COVID‐19 has greatly improved since despite the absence of antiviral drugs. This has been especially true in China when treating moderately ill cases, with the disease in its early stages, via auxiliary therapy combined with Traditional Chinese Medicine.^26^ COVID‐19 patients have since benefitted from better understanding of the pathological changes and laboratory parameters of the disease. The death rate declined thereafter. In this report, there were no deaths among 52 cases analyzed, which included 7 severely and critically ill patients (Table 1A and B).

### 4.1 Hematological parameters are indexes of recovery/ prognostication of COVID‐19

Clinical observation found that the lymphocytes and T‐cell subsets were reduced in most of the COVID‐19 patients and were associated with disease severity.^13^, ^27^ In this report, it was found that CD3+, CD4+, CD8+ T cells, total lymphocytes, RDW, eosinophils, and basophils in pre‐treatment were significantly lower than in post‐treatment (*P* = .000, .000, .000, .012, .04, .000, and .001, respectively). Our results are similar to previous reports which merit a clinical significant in monitoring the severity of COVID‐19.^8^, ^13^, ^23^, ^24^, ^25^, ^27^ While the inflammatory indexes neutrophils, neutrophil/lymphocyte ratio (NLR), and CRP were significantly higher in pre‐treatment than in post‐treatment (*P* = .004, .011, and .017, respectively), which have been noticed and reported previously in COVID‐19 patients. Thus, these parameters are considered useful indicators for the management of COVID‐19.^8^, ^11^ Hemoglobin and RBC were significantly reduced post‐treatment (*P* = .032, .026), suggesting anemia occurred in patients during the treatment course. Increased PLT was observed post‐treatment although not significantly (*P* = .183) (Table 2). The reduction of lymphocytes and T‐cell subsets are believed to have been associated with the consumption and apoptosis of the cells when aggregated at the inflammatory sites fighting against the virus; alternatively, they could have been depleted by the over‐secreting of cytokines and inflammatory proteins.^8^, ^14^, ^27^, ^28^, ^29^, ^30^ The prognosis is poor if the lymphocytes and T‐cell subsets are persistently low and the CD4+/CD8+ ratio is high.^29^ In our observation, the post‐treatment lymphocytes and T‐cell subsets recovered, and all 52 cases were cured (Table 1). Higher neutrophils, NLR, and CRP levels were observed before treatment and were subsequently significantly reduced after treatment (*P* = .004, .011, and .017, respectively) (Table 2). The functions of the T‐cell subpopulation in infection and immune response were reported previously.^29^, ^31^, ^32^, ^33^, ^34^

### 4.2 Alterations in hematological parameters are more common and significant in cases of severely and critically ill patients

We did not perform statistical analysis comparing the parameters between severely and critically ill patients and moderately ill patients due to too few cases of severely and critically ill patients. The T‐cell subsets (CD3+, CD4+, CD8+) and total lymphocytes in severely and critically ill patients were apparently lower than in moderately ill patients. Neutrophils, NLR, PCT, and CRP levels were significantly higher in severely and critically ill patients than in moderately ill patients (*P* = .005, .002, .049, and .002, respectively) (Table 3). Alterations in laboratory parameters were associated with the severity of COVID‐19.^35^, ^36^ The causes of reduction in basophils are unclear; it could be due to consumption while fighting against the virus at infected sites similar to lymphocetes.^35^ Studies found that eosinophils were also reduced in severely ill COVID‐19 patients;^14^ however, our data did not show this phenomenon possibly as a result of the small number of severely and critically ill cases observed in the study.

Interestingly, we found that in severely and critically ill patients, there was a positive correlation among PT, ALT, and AST values indicating multi‐organ damage occurred from the virus attack as they were mentioned in other observations.^37^ Thus, they could be new useful parameters in evaluation of the treatment response and prognosis for COVID‐19.

### 4.3 Patients above 50 years of age exhibited more significant reduction in lymphocytes and T‐cell subsets

Further analysis revealed that CD3+, CD8+, total lymphocytes, platelets, and basophils in patients older than 50 were significantly lower than in patients younger than 50 (*P* = *P* = .049, .018, .019, .010, and .039, respectively), while neutrophils, NLR, CRP level, and RBC RDW in patients older than 50 were significantly higher than in patients younger than 50 (*P* = .0191, .015, .009, and .010, respectively) (Table 4). These results were similar to previous reports.^14^, ^28^, ^29^, ^38^, ^39^, ^40^, ^41^ The reduction of T‐cell subsets and the high CD4+/CD8+ T‐cell ratio are associated with disease severity; older‐aged cases tend to be more severe; thus, more lymphocytes would be consumed or severely impaired in the immune reaction. Again, higher RBC RDW suggests the occurrence of anemia in those patients.

## 5 CONCLUSIONS

Our study results further confirmed that hematological parameters are important in the effort toward better understanding of the clinical pathologic changes in patients of COVID‐19 and the improvement of the guidance of treatment and prognostication.

## AUTHOR CONTRIBUTIONS

Juanying Liang and Shaoyun Nong collected data and clinical information; Liejun Jiang, and Xiaowei Chi performed data analysis; Dewu Bi, Jun Cao, Lida Mo, and Xiaolu Luo performed the routine analysis; Huayi Huang was responsible for conception and writing.
